# **Urbanization, socioeconomic status, and exposure to PM**_**2.5**_ **, associated with township-based cerebrovascular disease (CBD) mortality**

**DOI:** 10.1371/journal.pone.0324070

**Published:** 2025-06-06

**Authors:** Wen-Yu Lin, Ping-Yi Lin, Chih-Da Wu, Wen-Miin Liang, Hsien-Wen Kuo

**Affiliations:** 1 Institute of Environmental and Occupational Health Sciences, National Yang Ming Chiao Tung University, Taipei, Taiwan; 2 Resource Circulation Administration, Ministry of Environment, Taipei, Taiwan; 3 Department of Nursing, Hungkuang University, Taichung, Taiwan; 4 Department of Medical Research, China Medical University Hospital, Taichung, Taiwan; 5 Department of Geomatics, College of Engineering, National Cheng Kung University, Tainan, Taiwan; 6 National Institute of Environmental Health Sciences, National Health Research Institutes, Miaoli, Taiwan; 7 Innovation and Development Center of Sustainable Agriculture, National Chung Hsing University, Taichung, Taiwan; 8 Research Center for Precision Environmental Medicine, Kaohsiung Medical University, Kaohsiung, Taiwan; 9 Department of Health Services Administration, China Medical University, Taichung, Taiwan; 10 School of Public Health, National Defense Medical Center, Taipei, Taiwan; Manchester Metropolitan University, UNITED KINGDOM OF GREAT BRITAIN AND NORTHERN IRELAND

## Abstract

**Background/Objective:**

Previous research has shown an association between socioeconomic status (SES) and mortality, particularly in chronic diseases. However, limited studies simultaneously examined the relationship between urbanization, SES, exposure to PM_2.5_, and cerebrovascular disease (CBD) mortality at a township level from 2011 to 2020 in Taiwan.

**Methods:**

Township-level SES data (percentages of low-income and education with college and above) and seven levels of urbanization from 2011 to 2020 were obtained from data sources in Taiwan’s central government. Age-standardized CBD mortality rates in 358 townships were calculated using the Geographic Information System (GIS) provided by the Research Center for the Humanities and Social Sciences (RCHSS) at Academia Sinica. Exposure to PM_2.5_ concentration was estimated using a combination of land-use regression and Ordinary Kriging to enhance the robustness of PM_2.5_ concentration estimates at the township level. Panel regression and structural equation modeling (SEM) was employed to analyze the association between urbanization, SES, exposure to PM_2.5_, and township-based CBD mortality rates.

**Results:**

There are significant differences in SES variables and exposure to PM_2.5_ among townships with seven levels of urbanization (P < 0.001). Even after controlling for other covariates (SES and PM_2.5_ concentration) through multivariate analysis, the associations between CBD mortality rates and urbanization areas persisted. SEM analysis revealed a negative correlation between age-standardized CBD mortality rate and education levels (β = −0.22), but a positive correlation with the proportion of low-income individuals (β = 0.41). There was no significant association between exposure to PM_2.5_ and CBD mortality. The panel regression analysis revealed that socioeconomic variables had different effects on CBD mortality rates across the three models (pooled ordinary least squares, fixed-effects, and random-effects) in both urban and rural areas. Notably, the level of urbanization was observed to modify the relationship between socioeconomic variables and CBD mortality rates.

**Conclusion:**

Our findings suggest that township-based CBD mortality is significantly associated with SES variables and levels of urbanization, despite a reduction in CBD mortality from 2011 to 2020. Therefore, targeted intervention programs should be implemented to reduce CBD mortality in different levels of urbanization, particularly in remote townships. It is necessary to assess the disparities in socioeconomic status to achieve a fair allocation of resources at the township level.

## Introduction

Cerebrovascular disease (CBD), including stroke, is a leading cause of mortality and disability worldwide [1,2]. Numerous studies have investigated the risk factors and determinants of CBD, including traditional cardiovascular risk factors such as hypertension, diabetes, and smoking [3,4]. However, there is limited research exploring the association between urbanization, socioeconomic status (SES), exposure to fine particulate matter (PM_2.5_), and CBD mortality at the regional level. Understanding the impact of urbanization, SES, and PM_2.5_ on CBD mortality is crucial for public health planning and interventions. Urbanization is often accompanied by changes in lifestyle, pollution levels, and socioeconomic disparities, which can influence health outcomes [5]. SES, which encompasses various socioeconomic factors such as income, education, and occupation, has been recognized as a determinant of health disparities [6,7]. Salonen (1982) linked urban living to a higher risk of cerebral stroke in men, and a lower level of education to an increased risk of cancer [8]. Additionally, exposure to PM_2.5_, a harmful air pollutant primarily emitted from industrial and vehicular sources, has been linked to adverse cardiovascular outcomes, including stroke [9,10]. Cardiovascular and stroke mortality rates were associated with neighborhood deprivation, while circulatory disease mortality rates were linked to long-term ambient pollution (relative risk (RR)=1.06, 1.00 to 1.13) and proximity to traffic (RR = 1.40, 1.08 to 1.81). Therefore, Finkelstein et al. [11] concluded that subjects residing in deprived neighborhoods had higher exposure to ambient particulate and gaseous pollutants as well as traffic.

According to the study by Lin et al. [12] in Taiwan, individuals residing in highly urbanized areas (level 1) had the highest stroke prevalence (2.49%). However, given the overall declining trend in stroke prevalence, it remains challenging to determine whether lower urbanization levels directly contribute to a reduction in stroke prevalence. After adjusting for other stroke risk factors, the study still identified the level of urbanization as a significant contributing factor to the overall prevalence of strokes. Moreover, most previous studies have not comprehensively accounted for multiple socioeconomic variables and PM_2.5_ exposure when examining their association with stroke. One possible explanation is that urbanized areas tend to have higher population densities, increased environmental pollution, and greater exposure to PM_2.5_, all of which are associated with an increased risk of cerebrovascular diseases. In contrast, rural areas may face challenges such as limited healthcare access, lower SES, and lifestyle factors that also contribute to stroke risk. Additionally, prior research has often overlooked the combined effects of multiple socioeconomic factors and PM_2.5_ exposure on stroke incidence and mortality. Therefore, by examining the interplay between urbanization, SES, exposure to PM_2.5_, and regional-based CBD mortality, this study tries to fill the gap in the literature and provide valuable insights into the complex relationship between these factors. Understanding these relationships can help policymakers and public health authorities develop targeted strategies to reduce CBD mortality and promote health equity. Moreover, investigating regional variations in CBD mortality and its association with urbanization, SES, and PM_2.5_ exposure can identify high-risk areas and vulnerable populations. This knowledge can guide resource allocation, prioritize interventions, and inform urban planning and environmental policies aimed at improving population health and reducing health inequalities [13]. Overall, this study’s motivation lies in the need to comprehensively understand the multilevel determinants of CBD mortality and their interplay at the township level. By exploring the relationships between urbanization, SES, exposure to PM_2.5_, and CBD mortality, we can advance our knowledge of the complex interactions between social, environmental, and health factors and inform evidence-based interventions to reduce CBD mortality and health inequality.

## Materials and methods

### Measurement of socioeconomic status (SES) variables, urbanization, and PM_2.5_

All of the Education level (Proportion of the population with a university degree or above) is obtained from the Government Open Data Platform. The data provided by the Ministry of the Interior contains information on the educational attainment of the population aged 15 and above at the township and village levels. In this study, the number of individuals with a university degree or above is divided by the total population of each township area to obtain the proportion of individuals with a university degree or above in each area. The population data for each township area is sourced from the Ministry of the Interior’s Department of Household Registration. The proportion of low-income households in each township area is obtained from the Ministry of Health and Welfare’s Statistics Department. The population data for each township area is sourced from the Ministry of the Interior’s Department of Household Registration. The number of low-income households in each area is divided by the total population of that area to obtain the proportion of low-income households. Lastly, the median income tax is derived from the statistical analysis tables of counties, cities, townships, and villages in the Statistical Monograph published by the Fiscal Information Agency, Ministry of Finance. The median income tax for each township or city area is calculated, and the unit is in thousands of New Taiwan Dollars (NTD). All data from the governmental website were retrieved from 2011 to 2020.

Degree of Urbanization is derived from the “Taiwan Social Change Survey,” which was initiated by the Ministry of Science and Technology, National Science Council in 1983. This survey follows the principle of conducting surveys every five years. In 2001, the “Taiwan Social Change Survey” joined the International Social Survey Program (ISSP) and began conducting annual surveys in Taiwan synchronized with other member countries from 2002 onwards. Additionally, since 2006, the “Taiwan Social Change Survey” has participated in the East Asia Social Survey (EASS), which includes Taiwan, South Korea, Japan, and China. The EASS conducts a joint thematic survey every two years. The survey employs strict quality control and comparison measures for interviewer surveys. Based on six categories including the percentage of the commercial population, industrial population, population aged 15–64, population aged 65 and above, population with tertiary education or higher, and population density, the survey classifies the data. The clustering method used to measure the differences within the same variable is Ward’s Minimum Variance Method, with the squared Euclidean distance as the measurement distance method. As a result, the 358 townships in Taiwan are divided into seven clusters: Cluster 1: Metropolitan core; Cluster 2: Industrial and commercial areas Cluster; 3: Emerging towns Cluster; 4: Traditional industry towns Cluster; 5: Low-developed townships Cluster; 6: Aging townships Cluster; 7: Remote townships.

The initial model for the annual average PM_2.5_ concentration utilized a Hybrid Kriging/Land-use Regression (LUR) approach. This allowed us to simulate the concentration and identify crucial spatial explanatory variables [14,15]. Briefly, using ArcGIS Pro 2.6, various land-use characteristics within different-sized circular areas (ranging from 25, 50, 100, 250, 500, 750, 1000, 1250, 1500, 1750, 2000, 2500, 3000, 4000, to 5000 meters) surrounding the sampling points along the flight paths were statistically calculated. These characteristics included the number of restaurants, temples, residential areas, industrial zones, road density, vegetation distribution, and more. In addition, three-dimensional spatial parameters within three-dimensional circular areas (with radii of 25, 50, 75, 100, 125, and 150 meters) were also considered. These land-use variables were used as explanatory variables in the linear model construction, with the concentration information at the sampling points as the dependent variable. A stepwise variable selection method was applied, with p-values smaller than 0.1 and larger than 0.3 serving as the criteria for the inclusion and removal of variables. The direction of the correlation between the selected variables and the predicted target was examined during the model construction (e.g., the proportion of artificial surfaces should be positively correlated with air pollution particles, while environmental greenness and vegetation coverage should exhibit a negative correlation). To avoid errors in parameter estimation caused by collinearity, the variance inflation factor (VIF) was used as a criterion. Variables with VIF values greater than 3 were excluded. The LUR model was then used to establish the annual average PM_2.5_ concentration for each township area. The validation of the model included overfitting and robustness assessments, which involved (1) holdout validation, (2) 10-fold cross-validation, and (3) external data validation.

### Measurement of outcome

The standardized mortality rate (SMR) for CBD is derived from the Center for GIS, RCHSS, Academia Sinica, and their Taiwan SMR Map. The population data on the website is obtained from the population statistics of various counties and townships in the Social and Economic Database of the Ministry of the Interior, with the standard population being the WHO 2000 World Population. The standardized mortality rate is calculated using direct standardization. The calculation method is as follows: First, calculate the mortality rate for each age group. For each year, gender, township area, and specific disease, there are 18 age groups (0–4 years, 5–9 years, 10–14 years,..., 80–84 years, and 85 years and above). Calculate the crude mortality rate for each age group (number of deaths in that age group/ population in that age group). Multiply the crude mortality rate for each age group by the age-specific population ratio of the 2000 world standard population, which gives the SMR for each age group. Finally, sum up the SMR for each age group, multiply by 100,000, and obtain the age-standardized SMR for each year, gender, and township area level. All data were retrieved from various datasets available on the website. Please review them under ‘Data Availability’ later.

### Statistical method

All analyses were performed using SPSS package version 24. Univariate analysis was employed to describe the levels of urbanization and age-standardized CBD mortality rates. Multiple regression analysis was conducted, adjusting for socioeconomic variables, to examine the relationship between urbanization levels and age-standardized CBD mortality rates. Panel regression analysis was conducted using three models, pooled ordinary least squares (OLS), fixed effects (FE), and random effects (RE), to assess the association between socioeconomic variables and age-standardized CBD mortality rates in both urban and rural areas. The general form of the regression model was specified as: CBD mortality*it* = β₀ +  β₁Low income*it* +  β₂Education*it* + β₃PM_2.5_*it* + ε*it*, where β represents the regression coefficients and εit is the error term. The pooled OLS model used ordinary least squares estimation. The FE and RE models were estimated using maximum likelihood (ML) or restricted maximum likelihood (REML), depending on model specification. A structural equation model (SEM) was employed to investigate the mediating effect of urbanization on the relationship between low income, education, exposure to PM_2.5_, and age-standardized CBD mortality rates.

## Results

Table 1 presents the average age-standardized CBD mortality rates, along with socioeconomic variables and PM_2.5_ levels across 349 townships in Taiwan from 2011 to 2020. There is significant variation in these variables over the ten-year period. The average age-standardized CBD mortality rate was 34.41 per 100,000, with a range from 18.54 to 221.14 per 100,000. The average percentage of low-income households was 2.08%, and the average percentage of townships with a high education level was 19.08%. Similarly, the average PM_2.5_ concentration was 24.09 µg/m³, ranging from 4.80 to 48.03 µg/m³.

**Table 1:** Average age-standardized mortality rates for CBD, socioeconomic variables, and PM_2.5_ levels across 349 townships in Taiwan (2011–2020).

	Mean ± S.D. (Min-Max)
Age-Standardized CBD mortality (1/10^5^)	34.41 ± 18.54 (18.54-221.14)
Low-income (%)	2.08 ± 2.02 (0.02-20.04)
Education (%)	19.08 ± 8.24 (4.74-75.80)
PM_2.5_ (μg/m^3^)	24.09 ± 8.36 (4.80-48.03)

The association between different levels of urbanization and the proportions of low-income households, the proportion of individuals with a college degree or above, and exposure to PM_2.5_ concentration are presented in Table 2. The results indicate significant differences among regions with varying degrees of urbanization and all variables (P < 0.001). Specifically, lower levels of urbanization are associated with a higher proportion of low-income households. Conversely, urbanization is positively correlated with the proportion of individuals with a college degree or above and annual tax payments. This means that as the level of urbanization increases, the proportion of individuals with a college degree or above also tends to increase. However, in the region with the highest level of urbanization, the exposure to PM_2.5_ concentration is the highest (27.38 μg/m^3^), and there is a negative correlation between levels of urbanization and PM_2.5_ concentration.

**Table 2:** Urbanization level associated with low income, education, and exposure to PM_2.5_.

	Low income (%)	Education (%)	PM_2.5_ (µg/m3)
**Metropolis Core**	1.72 ± 1.04	32.67 ± 8.48	27.38 ± 7.59
**Industrial and commercial urban**	1.41 ± 0.55	28.29 ± 6.56	23.18 ± 8.57
**Emerging towns**	1.25 ± 0.72	22.40 ± 6.04	26.29 ± 7.54
**Traditional industrial towns**	1.09 ± 0.47	19.61 ± 4.30	24.20 ± 6.64
**Low-level development townships**	2.27 ± 1.50	14.56 ± 3.65	23.96 ± 9.23
**Aging townships**	1.85 ± 1.61	13.04 ± 3.14	24.45 ± 7.75
**Remote townships**	6.51 ± 3.60	10.06 ± 3.03	17.24 ± 6.66

There are significant differences in all variables across different levels of urbanization.

Table 3 shows the difference between the levels of urbanization in different counties and the age-standardized CBD mortality rate. Urbanization levels are divided into seven categories, with the remote townships used as the reference value. The results indicate a negative correlation between levels of urbanization and the age-standardized CBD mortality rate. In the crude model, it was found that the Metropolis Core area had a higher CBD mortality rate than more remote areas by 39.457 deaths per 100,000 people. As the level of urbanization decreases, there is an increasing trend in the age-standardized CBD mortality rate. In the adjusted model, controlling for other covariates such as the percentage of education with college or above, the percentage of low-income individuals, and exposure to PM_2.5_, the trends of CBD mortality rate associated with urbanization areas remain. However, the regression coefficients show a decreasing trend compared to the crude model.

**Table 3:** Urbanization level associated with age-standardized CBD Morality.

Area	Crude model	Adjusted model^a^
Metropolis Core	−39.457** (1.314)	−22.354** (1.851)
Industrial and commercial urban	−36.260** (1.163)	−20.206** (1.647)
Emerging towns	−31.993** (1.035)	−17.770** (1.483)
Traditional industrial towns	−29.546** (1.122)	−16.069** (1.514)
Low-level development townships	−28.499** (0.992)	−19.324** (1.274)
Aging townships	−29.537** (1.156)	−20.022** (1.439)
Remote townships	Reference	Reference

^a^Adjusted for the percentage of education with college above, percentage of low-income, and exposure to PM_2.5_; *p < 0.01; **p < 0.001.

**Fig 1:**
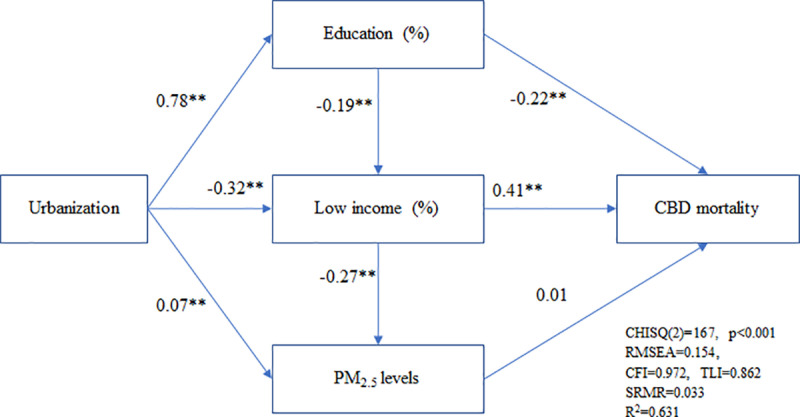
The structural equation model depicted age-standardized cerebrovascular disease (CBD) mortality associated with urbanization, education, low income, and exposure to PM_2.5_; *p < 0.01; **p < 0.001.

Fig 1 presents the results of an SEM analyzing the relationships between levels of urbanization, the proportion of individuals with a college degree or above, the proportion of low-income individuals, exposure to PM_2.5_, and the age-standardized CBD mortality rate at the township level. The results indicate levels of urbanization are positively correlated with the proportion of individuals with a college degree or above but negatively correlated with the proportion of low-income individuals. This indicates that townships with lower levels of urbanization have a higher proportion of low-income individuals (β = −0.32), while counties with higher levels of urbanization have a higher proportion of individuals with a college degree or above (β = 0.78). The proportion of individuals with a college degree or above is negatively correlated with the age-standardized CBD mortality rate (β = −0.22) and the proportion of low-income individuals (β = −0.19). This implies that higher proportions of individuals with a college degree or above are associated with lower age-standardized CBD mortality rates and lower proportions of low-income individuals. The proportion of low-income individuals is positively correlated with the age-standardized CBD mortality rate (β = 0.41) but negatively correlated with exposure to PM_2.5_ (β = −0.27). This indicates that townships with higher proportions of low-income individuals have higher age-standardized CBD mortality rates but lower exposure to PM_2.5_. However, there is no significant correlation between exposure to PM_2.5_ and the age-standardized CBD mortality rate. In summary, urbanization acts as a mediating factor in the relationship between socioeconomic variables and CBD mortality, but not in relation to exposure to PM_2.5_. The SEM model has examined the fitness of the goodness-of-fit criteria.

Table 4 utilizes three models (Pooled OLS, fixed-effect (FE), and random-effect (RE) in Panel regression to examine the heterogeneity and temporal variations in age-standardized CBD mortality rates across different townships in Taiwan, classified into urban and rural areas based on the level of urbanization. The primary explanatory variables include the proportions of low-income households, the proportion of individuals with a college degree or higher education, and exposure to PM_2.5_ concentration. In Model 1, OLS, FE, and RE were employed, revealing significant disparities in CBD mortality rates attributed to the proportions of low-income households between the OLS and RE models. Specifically, as the proportions of low-income households increase, the CBD mortality rate also tends to rise. In Model 2, the proportion of individuals with a college degree or above was introduced as an additional variable. The outcomes consistently indicated a significant negative correlation between this variable and CBD mortality rates across all three models. Consequently, as the proportion of individuals with a college degree or above increases, the CBD mortality rate decreases. Obviously, the regression coefficient for the proportions of low-income households diminishes, although it still retains a significant impact. In Model 3, the variable of exposure to PM_2.5_ concentration was included. In urban areas, all three models exhibit a significant positive correlation with CBD mortality rates. However, in rural areas, only the FE model displays a significant positive correlation. Model 3 demonstrates the highest overall explanatory power (R^2^ = 0.25–0.31). Individuals with low income in rural areas exhibited a higher effect (β = 390.1 and β = 163.9) compared to those residing in urban areas (β = 203.6 and β = 86.4), as observed in both RE and FE models. Notably, the results indicate that levels of urbanization act as a modifier on socioeconomic variables and CBD mortality rates.

**Table 4:** Low income, education and exposure to PM_2.5_ levels associated with age-standardized cerebrovascular disease mortality using panel regression analysis.

		Pooled OLS	Fixed Effects	Random Effects	Pooled OLS	Fixed Effects	Random Effects	Pooled OLS	Fixed Effects	Random Effects
Urban level	Low income (%)	2.91**	1.15	2.53**	2.11**	0.93	1.92*	2.20**	0.86	2.04*
	Education (%)				−0.45**	−0.42**	−0.45**	−0.44**	−0.14	−0.41*
	PM_2.5_ (ug/m^3^)							0.10*	0.20**	0.10*
	R^2^	0.16	0.16	0.16	0.25	0.23	0.25	0.25	0.16	0.25
	rho		0.36	0.26		0.28	0.20		0.35	0.20
Rural level	Low income (%)	4.53**	0.77	3.25*	3.71**	0.40	2.45*	4.46**	1.64*	3.90**
	Education (%)				−0.60**	−0.71**	−0.75**	−0.44*	−0.18	−0.57**
	PM_2.5_ (ug/m^3^)							−0.04	0.30*	0.10
	R^2^	0.24	0.24	0.24	0.26	0.19	0.26	0.32	0.18	0.31
	rho		0.50	0.35		0.47	0.34		0.50	0.32
Total	Low income (%)	4.19**	0.87	3.11**	3.30**	0.59	2.43**	3.80**	1.40*	3.30**
	Education (%)				−0.54**	−0.53**	−0.58**	−0.50*	−0.13	−0.51**
	PM_2.5_ (ug/m^3^)							−0.02	0.30**	0.08
	R^2^	0.23	0.23	0.23	0.27	0.21	0.27	0.30	0.17	0.30
	rho		0.49	0.35		0.44	0.32		0.49	0.31

*p < 0.01; **p < 0.001.

## Discussion

This study indicated that township-based CBD mortality is significantly associated with SES variables and levels of urbanization, despite a reduction in CBD mortality from 2011 to 2020. The correlation between urbanization and SES variables found in this study is consistent with previous research. According to van Maarseveen’s study, there is a correlation between urbanization and increased educational achievement in children residing in urban regions [16]. This implies that urban areas generally provide superior educational prospects, resulting in a greater proportion of individuals with a higher socioeconomic status (SES). In addition, the negative correlation between urbanization and the proportion of low-income individuals aligns with studies by Jones et al. [17] and Wang et al. [18]. These studies found that urban areas generally have lower poverty rates and higher median household incomes compared to rural areas. This socioeconomic disparity is an important factor to consider when analyzing health outcomes, including CBD mortality. Obviously, the inverse relationship between education levels and CBD mortality rates observed in this study is consistent with previous research on the impact of education on health outcomes. A study by Cutler and Lleras-Muney [19] demonstrated that higher levels of education are associated with lower mortality rates and better overall health outcomes, including a reduced risk of CVD. Similarly, township-level per capita Gross Domestic Product (GDP) in China was positively associated with sufficient leisure time physical activity for both rural and urban areas and a healthy diet (p < 0.01 for urban areas), while negatively associated with none or moderate alcohol use (p < 0.01 for rural areas) [20]. The urban-rural disparity in healthy behaviors may contribute to differences in socioeconomic status, which in turn can impact CVD mortality rates. A systematic review examines how SES moderates the association between lifestyle factor combinations and adverse health outcomes. There are lower SES groups that may be disproportionately vulnerable to the effects of unhealthy lifestyle factors compared with higher SES groups via interactions with other factors associated with low SES (e.g., stress) or via accelerated biological aging [21]. However, it is challenging to illustrate the mechanisms and causal relationships between urbanization, SES, exposure to PM_2.5_, and township-based CBD mortality.

Previous studies have clearly indicated that SES influences mortality rates through access to healthcare, living conditions, education, and lifestyle choices. Individuals with higher SES typically enjoy better healthcare access, healthier lifestyles, improved living conditions, and greater social support, including preventive care, early diagnosis, and effective treatment for conditions like CVDs. Conversely, lower SES individuals often encounter barriers such as lack of insurance, higher out-of-pocket costs, and limited availability of healthcare services, leading to poorer health outcomes and higher mortality rates [22,23]. Urbanization often used as an indicator of social and economic development. While urban areas generally offer better healthcare infrastructure and opportunities, income disparities still result in significant health differences. Rural areas, however, face broader systemic challenges due to limited healthcare resources and economic opportunities, exacerbating the impact of income disparities on health. Addressing these issues requires targeted interventions that consider the unique challenges and opportunities in both urban and rural settings. Therefore, the interaction effect of SES and urbanization on CBD mortality results from the complex interplay between access to healthcare, environmental factors, social determinants, housing conditions, and economic inequality. In highly urbanized areas, lower SES individuals face compounded challenges that negatively affect their health and increase mortality rates [24]. Addressing these issues requires targeted interventions that consider both urban environmental conditions and socioeconomic disparities on CBD mortality. Additionally, PM_2.5_ and SES affect CBD mortality rates by influencing exposure and access to healthcare. Higher SES often means better living conditions and healthcare access, reducing PM_2.5_ exposure and improving health outcomes. Lower SES typically leads to higher PM_2.5_ exposure and limited healthcare access, increasing CBD mortality rates.

In our study, we utilized three models (Pooled OLS, Fixed Effects (FE), and Random Effects (RE)) in panel regression to investigate the relationship between socioeconomic variables and CBD mortality rates in urban and rural areas. The selection of the preferred model depends on the assumption regarding the correlation between the individual, cross-section error component (εt), and the regressors. If it is assumed that εt and the regressors are uncorrelated, the RE model is deemed appropriate. Conversely, if there is believed to be a correlation between εt and the regressors, the FE model may be more suitable. To determine the more appropriate model, the Hausman test was conducted [25]. Our findings indicated that socioeconomic variables had varying effects on CBD mortality rates across the three models in both urban and rural areas. Notably, the level of urbanization was observed to modify the relationship between socioeconomic variables and CBD mortality rates. Further research should aim to highlight the complex interactions and pathways between socioeconomic factors and health outcomes. Understanding the causal processes involved in these relationships is crucial for improving our knowledge and identifying effective preventive measures. By delving deeper into these interactions, we can gain valuable insights into the underlying mechanisms that link socioeconomic factors to CBD mortality. This knowledge can then inform the development of targeted interventions and policies aimed at addressing health disparities and promoting better health for all [26].

Although evidence has shown a positive association between the proportion of low-income individuals and CBD mortality rates [22,23], limited studies explored the correlation between health outcomes with SES variables and urbanization simultaneously. Based on SEM analysis, the study revealed a negative correlation between age-standardized CBD mortality rate and education levels (β = −0.22), but a positive correlation with the proportion of low-income individuals (β = 0.41). We suggested that the association between urbanization and CBD mortality is mediated by education levels and low-income households, indicating that urbanization influences CBD mortality through its impact on these socioeconomic factors. Furthermore, three-panel regression models also exhibited a significant positive correlation between the percentages of low-income individuals and education levels with CBD mortality rates. Whereas our findings cannot include lifestyles to examine the correlation with CBD mortality. It is well-known that a significant interaction was found between lifestyle and deprivation for all-cause and CVD mortality, but not for CVD incidence. The author concludes that wide combinations of lifestyle factors are associated with disproportionate harm in deprived populations [27]. The apparent disparity in access to and availability of medical resources has been identified as a contributing factor to low health literacy in rural or low socioeconomic townships. Overall, there was an increase in the prevalence of obesity, diabetes, and hypertension, with higher rates observed among low-income groups for each cardiovascular risk factor. Particularly noteworthy was the significant increase in the prevalence of physical inactivity, with the “lowest-income” group experiencing a relative percent increase of 71.1% [28]. Indeed, socioeconomic inequality can have a profound impact on health outcomes, leading to lower levels of health literacy and poorer health behaviors, which in turn can contribute to higher morbidity and mortality rates. Individuals with lower SES often face barriers to accessing quality healthcare, preventive services, and health education. Limited resources and opportunities can make it challenging to adopt healthy behaviors such as regular exercise, balanced diets, and avoiding harmful habits like smoking or excessive alcohol consumption. In a study conducted by Mahajan et al. [29], it was revealed that lacking awareness of symptoms was significantly associated with Hispanic ethnicity (OR, 1.89; 95% CI, 1.47–2.43) and lower education level (OR, 1.31; 95% CI, 1.09–1.58). Additionally, the study identified various sociodemographic subgroups that exhibited a higher risk of lacking awareness of myocardial infarction symptoms. Similarly, individuals with five high-risk characteristics (non-White, non-US born, low income, uninsured, and high school education or lower) showed an almost four-fold higher odds ratio (OR, 3.70; 95% CI, 2.43–5.62) of lacking awareness of all myocardial infarction symptoms. The study suggested that targeted public health interventions could potentially benefit specific sociodemographic subgroups characterized by reduced awareness [30].

Based on SEM findings, there appears to be no significant link found in this study between PM_2.5_ exposure and CBD mortality rates. Our findings, presented in Table 3, show that the effect of low-income households on CBD mortality was greater than the effect of PM_2.5_, as indicated by both fixed and random effects models. These results are consistent with previous studies suggesting that SES factors are a fundamental cause of health inequality. When SES and PM_2.5_ exposure were simultaneously included in the panel regression model, the positive effect of PM_2.5_ on CBD mortality appeared to be influenced by SES. This suggests that while PM_2.5_ is a significant factor, its impact on CBD mortality may be moderated by socioeconomic conditions. Possibly, it is crucial to acknowledge that township-level analyses may not fully capture the complex impact of PM_2.5_ on specific health outcomes. It is important to consider that our study assumes uniform PM_2.5_ exposure within townships, while there may be variations and heterogeneity in exposure levels across different townships. Furthermore, it is important to consider that township-level analyses may not account for other significant risk factors that contribute to CVD mortality. While this study focused specifically on the association between PM_2.5_ exposure and CBD mortality rates, it is essential to recognize that there are additional factors at play within townships that could influence CVD outcomes. These factors may include, but are not limited to, socioeconomic status (SES), lifestyle factors (such as smoking, alcohol consumption, physical activity, and dietary habits), access to healthcare, comorbidities (including diabetes, hypertension, hyperlipidemia, and heart disease), and genetic predispositions. The study assumes a homogeneous distribution of these potential factors across each township from 2010 to 2020. A comprehensive understanding of CVD mortality requires a more holistic examination that incorporates these potential confounding variables. Studies conducted by Brook et al. [31] and Pope [32] have reported associations between long-term exposure to PM_2.5_ and CVD, but their focus was on broader geographic scales or individual-level analyses. Therefore, strategies aimed at reducing the persistent health disparities gap may involve increasing coverage for preventive measures and promoting healthy lifestyle behaviors.

This study has several distinctive features that set it apart from previous research. Firstly, it adopts a township-level analysis, focusing on small geographical areas rather than individual-level data. By examining CBD mortality rates at the township level, this study provides a more comprehensive understanding of the impact of urbanization, SES, and exposure to PM_2.5_ on CBD. Unlike previous studies that primarily considered SES as a control variable, this study recognizes the importance of SES as a key determinant of CBD mortality. encompassing various socioeconomic factors such as income, education, and occupation, is considered a crucial factor in understanding health inequality. By exploring the association between SES and CBD mortality rates, this study contributes to a deeper understanding of the social determinants of health and their implications for CBD. Lastly, this study employs panel data analysis, which allows for the examination of temporal trends and variations over time. By analyzing data collected longitudinally from multiple time points, the study can assess the dynamic relationships between urbanization, SES, PM_2.5_ exposure, and CBD mortality. However, it is essential to acknowledge the limitations of this study. Firstly, the study design is based on township-level data and observational design, which may not capture individual-level variations and potential confounding factors (lifestyle factors, access to healthcare, comorbidities, and genetic predispositions). Also, it may be challenging to establish a causal relationship between the variables of interest. While panel data analysis can help in exploring temporal associations, it cannot definitively prove causality. Secondly, the data used for SES variables, PM_2.5_ exposure, and CBD mortality rates are subject to measurement errors and limitations inherent in data sources. Additionally, the study focuses on a specific geographic area, which may have ecological fallacy and limit the generalizability of the findings to other regions or countries. Future research should consider incorporating individual-level data and exploring additional factors that may contribute to CBD mortality rates. Longitudinal studies also provide valuable insights into the causal relationships between urbanization, SES, PM_2.5_ exposure, and CBD mortality. Moreover, investigating potential mediating or moderating factors could further elucidate the mechanisms underlying the observed associations.

## Conclusions

While the findings of this study align with existing literature regarding the relationships between urbanization, SES, PM_2.5_ exposure, and CBD mortality, we discovered a significant moderation effect of urbanization and SES on CBD mortality using a panel regression model. Additionally, we found the mediating effect of urbanization on CBD mortality using SEM. However, the absence of a significant association between PM_2.5_ exposure and CBD mortality rates emphasizes the need for further research to explore the underlying mechanisms and contextual factors involved. Addressing socioeconomic inequality and enhancing health literacy are pivotal in reducing CVD morbidity and mortality rates. It is essential to implement strategies that promote education, improve access to healthcare services, and foster supportive environments for healthy lifestyles. These efforts can help alleviate disparities and enhance overall cardiovascular health. Furthermore, this finding advances our understanding of these intricate relationships and establishes a basis for evidence-based interventions and public health policies.
